# Blood–CSF barrier dysfunction and acute psychosis: a case report

**DOI:** 10.1192/j.eurpsy.2026.10928

**Published:** 2026-07-31

**Authors:** E. Hoxha Jusufi, P. Sotiroski, M. Pislevski, M. Dimovska, V. Mitova, S. Bajraktarov

**Affiliations:** 1 University Clinic of Psychiatry, Faculty of Medicine, Ss. Cyril and Methodius, Skopje; 2 Psychiatric Hospital Demir Hisar, Demir Hisar, North Macedonia

## Abstract

**Introduction:**

Blood–CSF barrier dysfunction has been linked with psychosis and other psychiatric conditions. It can be assessed by elevated CSF/serum albumin ratio, IgG index, and total protein levels. Acute psychotic decompensation may arise as a consequence of immune activation, infection, or other stressors. Identifying such impairments during acute episodes may help clarify underlying mechanisms and guide treatment.

**Objectives:**

To illustrate the association between an acute transient psychotic episode and blood–CSF barrier dysfunction with suspected immunological CNS activity.

**Methods:**

A 29-year-old male presented to the emergency department with acute confusion, incoherent speech, disorientation to person, place, and time, paranoid ideation, cognitive slowing, neck pain, vomiting, and fever. He reported no chronic illnesses or regular medications. Neuroimaging (CT, MRI, EEG) was unremarkable. Nasal swab was positive for MRSA, serum CMV IgG was detected, and CSF analysis revealed elevated albumin, total protein, IgG, albumin coefficient, and IgG index, consistent with intrathecal IgG synthesis. Initial psychiatric evaluation confirmed acute psychotic decompensation; follow-up after one week showed complete remission.

**Results:**

The patient was treated with virostatic, antibiotic, corticosteroid and antipsychotic therapy, with marked clinical improvement within one week.

**Conclusions:**

Full remission following treatment targeting inflammation and infection supports the hypothesis that blood–CSF barrier hyperpermeability may precipitate acute psychotic episodes. These findings emphasize the importance of considering immunological screening in acute psychiatric presentations.

**Disclosure of Interest:**

None Declared

